# Molecular Identification of Falciparum Malaria and Human Tuberculosis Co-Infections in Mummies from the Fayum Depression (Lower Egypt)

**DOI:** 10.1371/journal.pone.0060307

**Published:** 2013-04-02

**Authors:** Albert Lalremruata, Markus Ball, Raffaella Bianucci, Beatrix Welte, Andreas G. Nerlich, Jürgen F. J. Kun, Carsten M. Pusch

**Affiliations:** 1 Institute of Tropical Medicine, University of Tübingen, Tübingen, Germany; 2 Institute of Human Genetics, Department of Medical Genetics, University of Tübingen, Tübingen, Germany; 3 Laboratory of Physical Anthropology, Department of Public Health and Paediatric Sciences, University of Turin, Turin, Italy; 4 Division of Paleopathology, History of Medicine and Bioethics, Department of Oncology, Transplants and Advanced Technologies in Medicine, University of Pisa, Pisa, Italy; 5 Institute of Pre- and Protohistory and Medieval Archaeology, Department of Early Prehistory and Quaternary Ecology, University of Tübingen, Tübingen, Germany; 6 Division of Paleopathology, Institute of Pathology, Academic Clinic München- Bogenhausen, München, Germany; St. Petersburg Pasteur Institute, Russian Federation

## Abstract

Due to the presence of the lake Quarun and to the particular nature of its irrigation system, it has been speculated that the Fayum, a large depression 80 kilometers south- west of modern Cairo, was exposed to the hazards of malaria in historic times. Similarly, it has been speculated that, in the same area, also human tuberculosis might have been far more widespread in the antiquity than in its recent past. If these hypotheses were confirmed, it would imply that frequent cases of co-infection between the two pathogens might have occurred in ancient populations. To substantiate those speculations, molecular analyses were carried out on sixteen mummified heads recovered from the necropolis of Abusir el Meleq (Fayum) dating from the 3^rd^ Intermediate Period (1064- 656 BC) to the Roman Period (30 BC- 300 AD). Soft tissue biopsies were used for DNA extractions and PCR amplifications using well-suited protocols. A partial 196-bp fragment of *Plasmodium falciparum* apical membrane antigen 1 gene and a 123-bp fragment of the *Mycobacterium tuberculosis* complex insertion sequence *IS6110* were amplified and sequenced in six and five of the sixteen specimens, respectively. A 100% concordance rates between our sequences and those of *P*. *falciparum* and *M. tuberculosis* complex ones were obtained. Lastly, concomitant PCR amplification of *P. falciparum* and *M. tuberculosis* complex DNA specific fragments was obtained in four mummies, three of which are ^14^ C dated to the Late and Graeco-Roman Periods. Our data confirm that the hydrography of Fayum was extremely conducive to the spread of malaria. They also support the notion that the agricultural boom and dense crowding occurred in this region, especially under the Ptolemies, highly increased the probability for the manifestation and spread of tuberculosis. Here we extend back-wards to ca. 800 BC new evidence for malaria tropica and human tuberculosis co-occurrence in ancient Lower Egypt.

## Introduction

Tuberculosis (TB) and malaria, two of the most ancient and deadly diseases of mankind, have ravaged human communities since the beginning of civilization and remain a major global health problem in the 21^st^ century [1], [2]. TB causes ill-health among millions of people each year and ranks as the second leading cause of death of adults from an infectious disease worldwide, after the human immuno- deficiency virus (HIV). The latest World Health Organization report indicate that there were almost 9 million new cases in 2011 and 1.4 million TB deaths [1].

Malaria is the 5^th^ cause of death from infectious diseases worldwide after respiratory infections, HIV/AIDS, diarrheal diseases and tuberculosis. In 2011, there were 1.2 million malaria deaths globally and malaria is recognized as the 2^nd^ leading cause of death from infectious diseases in Africa, after HIV/AIDS [2]–[3]. In many parts of sub-Saharian Africa, the geographic overlap between TB and malaria is extensive and co-infection with TB and malaria is likely to be common [4]–[5].

Several investigations aimed at tracing the origins and frequencies of malaria and TB back have been carried out on Egyptian mummified remains over the past 25 years [6]–[15]. Tuberculosis DNA has long been recognized in mummies from Upper Egypt dating to different historical periods [7]–[13]. *Mycobacterium tuberculosis* (MTB) complex DNA was successfully amplified and sequenced in populations from Thebes-West (Upper Egypt) dating to the New Kingdom (c.a. 1550-1000 BC) [7], [9]–[12]. Where additional characterization was possible, it was shown that human lineages of MTB complex were present: *M. tuberculosis* and possibly *M. africanum* but not *M. bovis* [12]. Molecular signatures of TB infection were found in individuals with and without macro-morphological typical signs of tubercular spondylitis and belonging to different age classes [9]. It was speculated that, in the affected populations, there was a relatively low life expectancy and that this may have resulted from a considerable proportion of chronic infections by various pathogenic organisms such as tuberculosis and other parasitoses (i.e. malaria, leishmaniasis, schistosomiasis and other worm infections) [9], [12].

Similarly, but to a lesser extent, also *Plasmodium falciparum* ancient DNA was identified in mummified skeletons from Thebes-West dating from the New Kingdom to the Late Period (1500-500 BC) [14] and in 18^th^ Dynasty royal mummies [15]. However, no cases of malaria and tuberculosis co-infections were reported. Furthermore, most of the studies carried out till the present day have focused on the impact of infectious diseases on ancient populations from Upper Egypt whereas less attention has been paid to populations from Lower Egypt. Stimulated by the notion that malaria and TB were rampant in 19^th^ and early 20^th^ century Fayum [16], the present studýs aim was to detect and characterize *P. falciparum* and MTB complex DNAs in sixteen mummified heads from Lower Egypt and, eventually, to verify, the existence of co-infections.

Here we not only extend back-wards to ca. 800 BC evidence for single malarial and mycobacterial infections but provide new evidence of falciparum malaria/tuberculosis co-occurrence in individuals from ancient Fayum (**Table 1**).

**Table 1:** Results of radiocarbon dating and molecular identification of infectious pathogens from sixteen Egyptian mummies.

ID no. OSUT	Period*	C^14^ dates	Age, y	Sex	*MTB*IS6110	§	*P.f. AMA1*	*P.f. MSP1*	§
1543	Roman	54–124 AD	12±30 mos.	M	**+**	TB	+	–	M
1554	Late	402–385 BC	30–40	F	**++**	TB	++	+	M
1564	Late/Hellenistic-Ptolemaic	358–204 BC	11±30 mos.	M	**+**	TB	++	+	M
1622	3^rd^ Intermediate	806–784 BC	20–30	M	**+**	TB	++	+	M
1585	Hellenistic/Ptolemaic	382–234 BC	20–30	F	**+**	TB	–	–	–
1608	3^rd^ Intermediate	801–777 BC	20–30	M	**–**	–	+	–	M
1643†	From 3^rd^ Intermediate to Roman	ND	20–30	F	**–**	–	+	–	M
1611	From 3^rd^ Intermediate to Roman	ND	20–30	F	–	–	–	–	–
1630	From 3^rd^ Intermediate to Roman	ND	40–60	M	–	–	–	–	–
1631	From 3^rd^ Intermediate to Roman	ND	adult	NA	–	–	–	–	–
1640	From 3^rd^ Intermediate to Roman	ND	30–50	M	–	–	–	–	–
1642	From 3^rd^ Intermediate to Roman	ND	20–30	M	–	–	–	–	–
1655	From 3^rd^ Intermediate to Roman	ND	20–30	F	–	–	–	–	–
1656‡	From 3^rd^ Intermediate to Roman	ND	20–30	M	–	–	–	–	–
1670	From 3^rd^ Intermediate to Roman	ND	30–40	M	–	–	–	–	–
1669	From 3^rd^ Intermediate to Roman	ND	4–5	NA	–	–	–	–	–

Abbreviations: ND, not determined; NA, not available; M, male; F, female; MTB, *Mycobacterium tuberculosis*; P.f., *Plasmodium falciparum*; positive amplification obtained in at least two independent experiments is indicated by “+”; results obtained in at least two independent experiments, in different laboratories, and by different investigators is indicated by “++”.

* Egyptian chronology: Third Intermediate Period: 1064- 525 BC; Late Period: 525-332 BC; Hellenistic Period: 332 BC-30 BC; Roman Period: 30 BC- AD 395. Egyptian chronology as given in Ikram S. (2003).

† Cause of death was due to a trauma caused by a sharp weapon on the parietal and occipital bone. No signs of healing are observable.

‡ Cause of death was due to a trauma caused by a sharp or semi-sharp weapon, in addition to an arrow injury. No signs of healing are observable.

§ infection identified in the analyzed individual; M, malaria; TB, tuberculosis.

## Results

**Figure 1:**
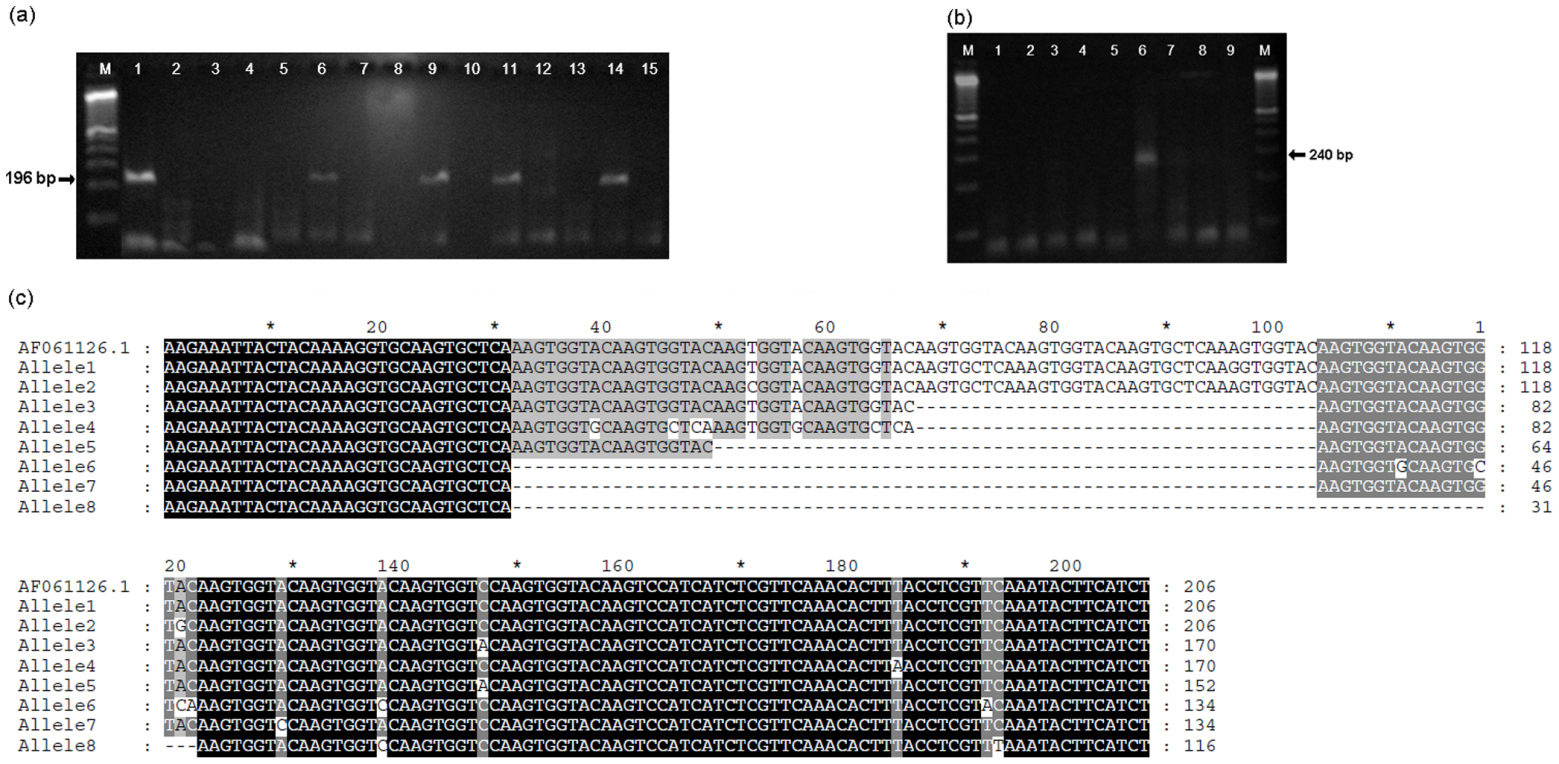
Identification of plasmodial DNA in Fayum mummies. (a) Amplification of partial apical membrane antigen 1 gene (*AMA1*). Lane M, 100- bp DNA ladder (NEB); lane 1, 1543; lane 6, 1554; lane 9, 1564; lane 11, 1622; lane 14, 1643; lane 15, PCR negative control; (b) Amplification of *MSP1* K1 allele. Lane M, 100- bp DNA ladder (NEB); lane 1, 1543; lane 2, 1554; lane 3, 1554; lane 4, 1622; lane 5, 1622; lane 6, 1564; lane 7, 1611; lane 8, 1643; lane 9, PCR negative control; (c) Nucleotide alignment of *Plasmodium* MSP1 K1 alleles amplified from mummy 1564 in comparison with *Plasmodium falciparum* isolate (accession no. AF061126). The variable central region of K1 allele types differ by the number and arrangement of repeat motifs. A total of 8 K1 type alleles were identified. Dashes represent gaps introduced to maximize the alignment.

Partial 196-bp *AMA1* gene was amplified in six of sixteen mummified heads (38%) (sample code numbers: 1543, 1554, 1564, 1608, 1622, 1643) (**Figure 1a** **&** **Table 1** **)**. The sequence alignment showed 100% homology to the corresponding reference sequence deposited in the NCBI with accession number FJ555864.

In addition, the parasite surface protein antigen *MSP1* allele was successfully genotyped in three of six malaria-positive mummies (50%) **(** **Figure 1b** **-** here 1564- **&** **Table 1** **).** More specifically, DNA extracts from the positive *AMA1* samples 1554, 1564 and 1622 showed amplification with K1 allele-specific primers whereas RO33 and MAD20 failed to amplify any of the 16 mummies tested (**Table 1**). This K1 allele from mummy 1564 was exemplary cloned into *E. coli* Top10 competent cells. Direct sequencing of the amplified clones using M13 primers showed eight unique K1 alleles with varying length in the repeat sequence **(** **Figure 1c** **)**.

**Figure 2:**
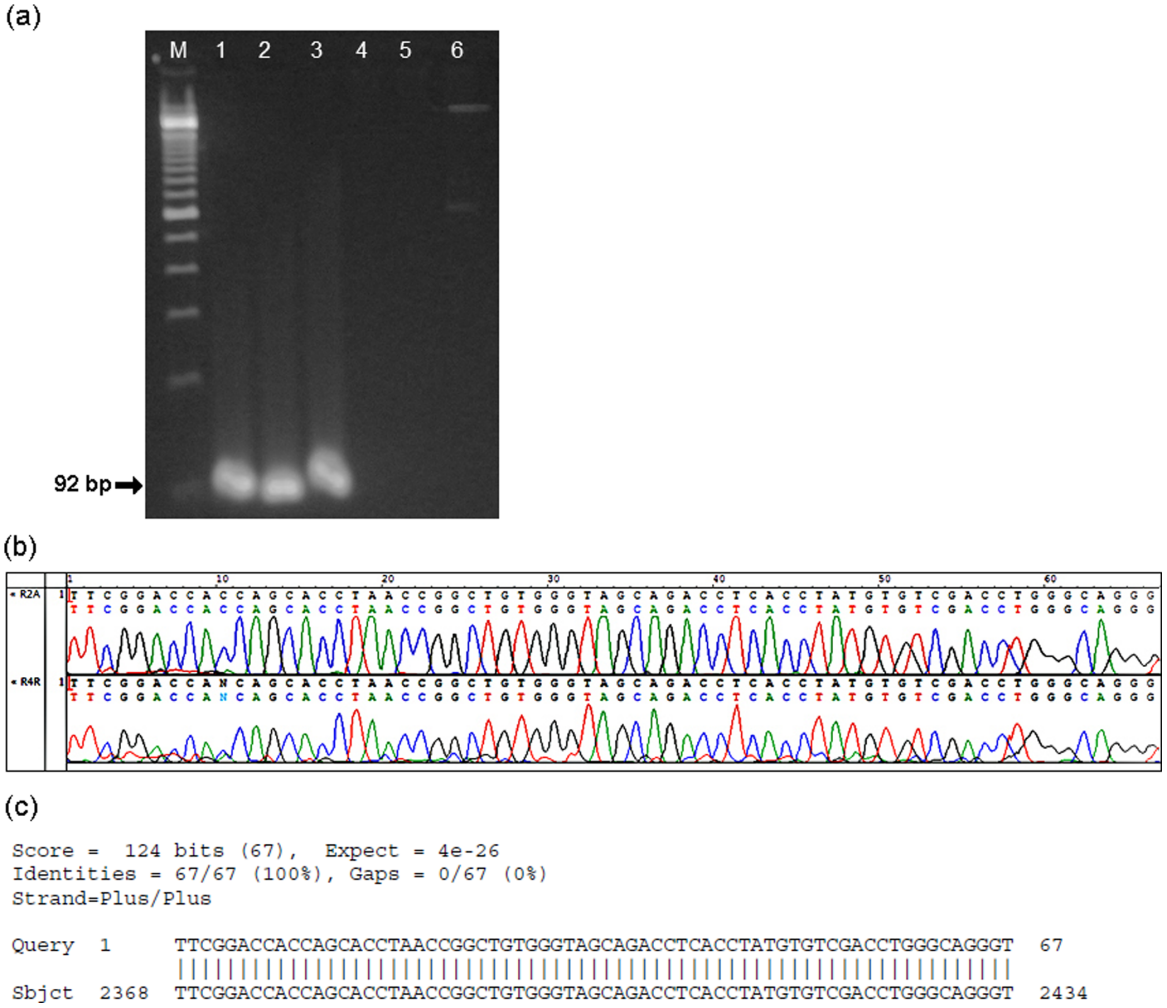
Identification of *Mycobacterium tuberculosi*s complex DNA in Fayum mummies. a. *M. tuberculosis* complex specific *IS6110* fragment (92-bp) amplified using nested primers (IS-3 and IS-4). Lane M, 100-bp DNA ladder (NEB); lane 1, 1554; lane 2, 1564; lane 3, 1622; lane 4, 1643; lane 5, PCR no template control; lane 6, extraction blank control; b. *M. tuberculosis* complex IS6110 (92-bp) sequence electropherogram and sequence similarity search by BLAST; c. Best hits include *Mycobacterium tuberculosis* GenBank ac. no. CP001642.1 showing 100% identity with an E-value of 4e-26.

Five out of the sixteen mummies (31%) represented by two pre-adolescents (1543, 1564), two young adults (1585, 1622) and one adult (1554), showed a specific 123-bp amplification product in the *IS6110* PCR reaction **(** **Figure 2a** **&** **Table 1** **)**. Direct sequencing of nested PCR fragments showed 100% homology to MTB complex DNA (GenBank accession number CP000642.1) (**Figure 2b, 2c** **)**. Spoligotyping, which distinguishes *M. bovis* from *M. tuberculosis* within the MTB complex, was not performed due to the limitations in sample size [12].

Apart from one case of single MTB complex infection (1585) and two cases of single malaria tropica infections (1608, 1643), the remaining four individuals (25%) (1543, 1554, 1564, 1622) show signatures of falciparum malaria and human tuberculosis co-infection (**Table 1**).

## Discussion

Since 1998 there have been no officially reported autochtonous cases of malaria in the Fayum Governatorate or elsewhere in Egypt [17]. Before the eradication of malaria from Egypt, high levels of the infection appeared to have been limited to certain parts of the country and to be strictly linked to its geology [16]. Due to the presence of the Birket Quarun, also known as Lake Moeris (which is fed by Bahr Yusuf), and to the particular nature of its irrigation system (i.e. a system of a network of smaller canals), the Fayum was highly exposed to the hazards of malaria in its recent past.

In the third quarter of the 19^th^ century and early 20^th^ century, especially after the third land reclamation carried out under Muhammad Ali’s reign (1805–1848), this area was reputed as the most malarial region outside the northern Delta and several cases of malarial and tubercular co-infections were registered in populations from this area [16].

Since the size of the lake and its water-level changed drastically over the past few millennia, it has been speculated that Fayum was even more conducive to malaria in historic times than in its recent past [16]. Following Sheidel [16], “*the manifestations of malaria that used to be endemic in the Mediterranean – quotidian, tertian and quartan fevers, associated with Plasmodium falciparum, vivax, and malariae – are explicitly attested in charms from Graeco-Roman Egypt. Hints at the occurrence of malaria can already be found in earlier periods, such as a warning in a temple inscription at Denderah not to leave home after sunset in the weeks following the inundation (when mosquitoes would have proliferated), reported strategies of mosquito evasion that are typical of malarial areas and, possibly, reference to the “disease of the three days”.*

*The same type of malaria was still common in Egypt in the 1930s, suggesting continuity in the long term. Comparative evidence makes it seem likely that the marshy Fayum would have been particularly exposed to endemic malaria”.*

Paleoenvironmental and paleoclimatic studies give us clues into the reconstruction of ancient Fayum’s landscapes [18]–[21]. Fluctuations in Neolithic times led to the success or demise of various agriculture or fishing communities on the lake shore [16], [18]–[21]. Before the Dynastic times the lake level was high (18–20 meters above the sea level (masl)) and covered most of the extensive depression. A general recession set in around the beginning of the Dynastic times (ca. 2880 BC). Around 2200 BC extensive dry conditions occurred (lower rainfalls) which determined Fayum’s lake receding levels. During the Old Kingdom (2663-2195 BC), the lake might have been as low as 2 meters below the sea level and no longer in free communication with the Nile [16]–[22].

In response to falling flood levels, the first land reclamation of Fayum occurred in the Middle Kingdom under the reigns of the 12^th^ Dynasty “engineering kings” [Senwosret II (1900-1880 BC) and Amenemhat III (ca 1880-1808 BC)] [16], [22], [23]. Although there’s no archaeological or textual evidence that sheds light on the exact extent of the works carried out in this period, this hydraulic project included the dredging of a branch of the Nile through the opening and widening of the Hawara canal, now called Bahr Yusuf, to allow waters to re-flow into the Fayum depression [16]–[18], [22].

Water flow was regulated to maintain the lake level at around 14 to 16 meters above the sea level in order to reclaim a vast area for agriculture. The locks that regulated the amount of water flowing into the depression are still located at the right side of the el-Lahun corridor [22]. A connected primitive canal system was created in order to irrigate the fields [16], [22].

At a time after 1800 BC, due to unexpected increase in flow discharge, the level of the lake raised considerably (ca 18 to 20 masl) and most of the areas occupied by the Middle Kingdom installations were drawn [16], [20]. The dam was breached and the Fayum depression became once more uninhabitable. The lake still stood at around the same level or even higher when Herodotus saw it in the mid-5^th^ century BC and apparently remained that way until early Ptolemaic times [16], [22].

The second large scale reclamation of the Fayum started under the Ptolemies [Ptolemy I Soter (310- 282 BC) and Ptolemy II Philadelphos (282-246 BC)] [22]. It probably took as long as 30 years to lower the level of the lake to two meters below sea level reaching the same level it had during the Old Kingdom.

The Lahun embankment (a five kilometers embankment from the northern side of the Lahun Gap at al-Lahun) constructed under the Ptolemies was used to divert the annual influx of Nile water. Lake Quarun, therefore, dropped to 5 meters below the sea level and the newly exposed land was colonized by Macedonian soldiers [16], [22]. The campaign of land reclamation appears to have started in the eastern area of the Fayum, in the meris of Herakleides, then continued in the southern area, corresponding to the meris of Polemon and, lastly, into the western area in the meris of Themistos [22].

This project added about 1.200 square kilometers of fertile land to the Fayum and attracted settlers, many of them from abroad. The development project led to an agricultural boom in the region with large settlement programs and the founding of many new towns. Hundreds of settlements were created [16].

During the Roman period, the Fayum became the granary of Egypt and Rome. The lake continued to shrink during the Roman Period (35 BC- AD 385) and declined from -7 meters in the 2^nd^ century AD to -17 or lower in the 3^rd^ century BC [22]. The whole canal system continued to be used, the agricultural productivity especially of wheat and barley was further optimised. Settlements formed with periods of high population density. The most reasonable guess for the overall population density in the Roman Period is about 200–300 people per km squared [16].

The reclamation of wetlands for cultivation and the creation of a system of canals for the irrigation of crops were apparently the key events which led to the exacerbation of the risk of malaria in this area.

Modern studies focusing on the linkage of land reclamation for cultivation and malaria’s spread in east Africa show that the cultivation of swampy depressions or swamp valley bottoms, as result of a rapid population and demand for more food, changes the local ecology [24], [25]. The wetlands are originally covered with natural papyrus, which limits the breeding of *Anopheles* (the vector) because of the dense vegetation and the oil layer. The elimination of papyrus and the reclamation of the swamps leads to an increase in temperatures promoting breeding of mosquitoes and, therefore, by increasing malaria transmission. If the irrigation system lacks efficient drainage, water accumulation is facilitated and, in turns this provides a breeding ground for mosquitoes. On the average, all malaria indexes (i.e. mosquitoes densities, biting rates, sporozoite rates) show to be much higher near cultivated swamps than near natural swamps [24], [25]. If the epidemiological relationship between increased malaria transmission and intensified crops cultivation is explored, the results show that the incidence of malaria is about was ten times higher in cereals-cultivation areas than in areas with less cereals (wheat pollens provide nutrition for larval anopheline mosquitoes). This implies that the intensity of crops cultivation is associated with exacerbated human risk of malaria. Also irrigation exposes non-immune populations in areas of unstable malaria transmission to high risk of acquiring the disease [24], [25].

For ancient Fayum’s populations, we hypothesize that reclamation of lands for cultivation and the constant contact between malaria’s vectors and human beings might have determined an increased risk of acquiring malaria. Furthermore, dense crowding of people may have been a significant factor also for the manifestation and spread of human tuberculosis and the risk of contracting concomitant infections might have been, therefore, augmented.

We confirm, using modern methods, that malaria and tuberculosis were endemic to the Fayum Depression from ca. 800 BC until the Roman Period (**Table 1** **)**. High frequencies of malaria and tuberculosis co-infections (4/16) (25%) were identified in pre-adolescents (2 cases) and young adults (2 cases) which appeared to be, as it occurs in modern populations, the most affected age-classes.

Modern epidemiology shows that, in areas of the world where tuberculosis and malaria are co-endemic, malaria infection affects severely ill TB patients who are already compromised by malnutrition, deprived immunity and disseminated disease. Interactions between TB and malaria have been demonstrated *in vitro* and *in vivo*: *Plasmodium falciparum* modulates *Mycobacterium tuberculosis* infection [26] and malaria has been shown to exacerbate mycobacterial infection [27].

The reasons for this are not completely explored but seem to involve parasite-parasite interaction and host-parasite interaction: malaria causes a further depression in immunity through a qualitative and quantitative defect in T lymphocytes, mainly CD8+ that are necessary for anti-mycobacterial response, and through a degeneration of the cytokine cascade [28], [29]. Moreover, the respiratory distress that is frequent during acute malaria both in children (due to metabolic acidosis) and adults (due to pulmonary edema and to acute respiratory distress syndrome) can worsen the respiratory efforts related to tuberculosis [30], [31]. Therefore, given the multiple interaction between malaria and TB, there’s a mutual effect in increasing mortality [32]. For the individuals we analyzed, we can hypothesize that *P. falciparum* infection played a significant role in increasing the incidence of reactivation of latent tuberculosis in adults or primary active tuberculosis in children and pre-adolescents, as it occurs in modern day populations.

On the basis of the results obtained so far, we feel confident to develop an ‘ancient pathogens genome project’ encouraged wholly by the improvement in sequencing technology well suited to ancient DNA research. This will allow us chronologically review pathogens evolution through their genomic sequences, compared with modern isolates and verify the evolutionary hypothesis about their origins. Many ongoing ancient DNA research aided by the ever improving next generation sequencing techniques in terms of throughput and analysis tools will provide a link to better insight into assessments of diseases in the past, their emergence and transmission.

## Materials and Methods

We analyzed soft tissue biopsies taken from 16 mummified heads belonging to the collection of the Institute of Pre- and Protohistory, Department of Early Prehistory and Quaternary Ecology Division of Paleoanthropology (Tübingen, Germany) (**Table 1**). Muscle biopsies (1 cm squared) from each head were taken from the inner neck region.

The heads (the rest of the bodies is missing) were recovered from the necropolis of Abusir el-Meleq in the Fayum Depression (Lower Egypt) at the beginning of the 20^th^ century [31], [32] and date from the 3^rd^ Intermediate Period (1064-656 BC) to the Roman Period (30 BC-300 AD) [23], [33].

### Authentication

Two ancient DNA laboratories dedicated for DNA extraction, PCR and Post-PCR analyses were established at the Department of Medical Genetics and Applied Genomics, University of Tübingen. No analyses with malaria or tuberculosis clinical specimens have ever been carried out in any of these laboratories. In addition, isolated replication analyses have been performed in another ancient DNA laboratory in Munich. DNA extraction, PCR and post-PCR analyses were performed separately in a dedicated room for each work. All reagents purchased were certified DNAase and RNAase-free molecular grade chemicals or were autoclaved if applicable. Disposable gloves, coats, and mouth masks were worn during all different procedures and changed frequently. Sterile aerosol protection filter tips (Biozym, Oldendorf Germany) were used in all experiments. Extraction blanks as well as negative PCR controls without template were included for every DNA extractions and PCR amplification. Replicating experiments were performed employing different laboratory space/equipment and enzyme/water aliquots and data were further verified by PCR-cloning and sequencing.

### DNA Extraction

Sampling from sixteen mummies (**Table 1**), DNA extraction and purification were performed following the protocol described earlier with modifications [34]. For DNA extraction, approximately 50–100 mg of tissue powder was placed in a 2 mL tube, 800 µl of DNA lysis buffer consisting of 10 mM Tris-HCL, pH 8,0, 10 mM NaCl, 2% SDS and 200 mg/mL proteinase K enzyme and 800 µl of phenol were added and mixed well. The solution was incubated overnight at 37°C incubator on a shaker. DNA was purified by chloroform and precipitated by addition of 1/10 volume of 3 M sodium acetate (pH 5.2) and an equal volume of chilled isopropanol. The pellets were washed with 70% ethanol and subsequently air-dried. All pellets were dissolved in nuclease-free water (Merck) and stored at -20C until further use.

### PCR Amplification for an Inhibitory Effect of Ancient DNA Extracts

In order to quantify the inhibitory effect of an ancient DNA extracts plasmid DNA with a known contemporary insert DNA was spiked with diluted ancient DNA extracts. M13 universal primer was used to amplify the plasmid insert in the presence of 1/10, 1/100 and 1/200 fold dilutions of ancient DNA extracts.

### Amplification and Sequencing of Plasmodium Falciparum DNA

To test for the presence of *Plasmodium falciparum* DNA in our ancient DNA extracts, PCR primers were designed to target small and partial fragments of apical membrane antigen 1 gene (*AMA1*) and polymorphic block 2 region of merozoite surface protein 1 gene (*MSP1*). *AMA1* is a protein expressed on the surface of merozoite stage *Plasmodium* parasite and is highly conserved across all apicomplexa parasites [35], [36]. This protein is involved in the merozoite invasion of red blood cells of the host and thus, essential for proliferation and survival of the malarial parasite inside the host. *MSP1,* another merozoite protein, exhibits extensive antigenic polymorphism among parasite strains/isolates [35], [36]. Based on sequence diversity analysis, *MSP1* gene is divided into 17 blocks: 7 highly variable blocks are interspersed with five conserved and five semi-conserved regions [36]. The MSP1 allelic variants fall under the 3 major types of allele families termed K1, RO33 and MAD20. Target genes locus were amplified in a 20 µl reaction volume containing 1X PCR buffer (Faststart, Roche), 1.5 mM MgCl2, 200 µM each of dNTPS, 2 µM of each primer (Sigma), 1 Unit of Faststart Taq polymerase (Roche), 2 µl of extracted aDNA and the reaction volume was made up to 20 µl by adding PCR grade water. The cycling conditions for *AMAI* PCR using GeneAmp ABI 2700 (Applied Biosystems) were: 94°C for 3 min, 45 cycles of: 94°C for 30 sec, 48°C for 30 sec, 72°C for 30 sec and the final extension at 72°C for 5 min. For *MSP1* the same cycling condition was used except the annealing temperature, which was at 55°C for 30 sec for all the three allelic families of *MSP1* (K1, MAD20 and RO33).

*AMA1* and *MSP1* gene fragments were amplified in a 20 µl reaction volume containing 1x PCR buffer (Faststart, Roche), 1.5 mM MgCl2, 200 µM each of dNTPS, 2 µM of each primer (Sigma), 1 Unit of Faststart Taq polymerase (Roche), DNA, and the reaction volume was made up to 20 µl by adding PCR grade water. The cycling conditions for *AMAI* PCR using GeneAmp ABI 2700 (Applied Biosystems) were: 94°C for 3 min, 45 cycles of: 94°C for 30 sec, 48°C for 30 sec, 72°C for 30 sec and the final extension at 72°C for 5 min. For *MSP1* the same cycling condition were applied but using 55°C as the annealing temperature for the three *MSP1* alleles K1, MAD20 and RO33 (**Table 2**).

**Table 2:** Details of primer pairs used in this study.

Primer name	Target	Annealing	Sequence (5′-3′)
AMA1-f	Plasmodium AMA1	48°C	CCCGCACCACAAGAACAAAAT
AMA1-r	Plasmodium AMA1		ACTGGACATTTCCCTGATGG
Msp1K1-f	Plasmodium MSP1 K1 alleles	55°C	GAAGAAATTACTACAAAAGGTG
Msp1K1-r	Plasmodium MSP1 K1 alleles		CACTTTACCTCGTTCAAATACTTCATCT
Msp1RO33-f	Plasmodium MSP1 RO33 alleles	55°C	TAAAGGATGGAGCAAATACTCAAGTTGTTG
Msp1RO33-r	Plasmodium MSP1 RO33 alleles		CATCTGAAGGATTTGCAGCACCTGGAGATC
Msp1MAD20-f	Plasmodium MSP1 MAD20 alleles	55°C	AAATGAAGGAACAAGTGGAACAGCTGTTAC
Msp1MAD20-r	Plasmodium MSP1 MAD20 alleles		ATCTGAAGGATTTGTACGTCTTGAATTACC
P1	Mycobacterium IS*6110*	68°C	CTCGTCCAGCGCCGCTTCGG
P2	Mycobacterium IS*6110*		CCTGCGAGCGTAGGCGTCGG
IS-3	Mycobacterium IS*6110*	58°C	TTCGGACCACCAGCACCTAA
IS-4	Mycobacterium IS*6110*		TCGGTGACAAAGGCCACGTA

*AMA1* and *MSP1* PCR products were cleaned by ExoSAP-IT (USB) and directly used for cycle- sequencing with BigDye terminator v3.1 chemistry (Applied Biosystems). *MSP1* PCR products were cloned using TOPO TA cloning kit (Invitrogen) and transformed into *E. coli* Top10 competent cells. Colonies were PCR-screened and sequenced with M13 universal primers (Eurofins MWG Operon).

### Amplification and Sequencing of *Mycobacterium tuberculosis* DNA

PCR amplification was performed using primers P1 and P2, recognizing a 123-bp fragment of the IS6110 sequence of *M. tuberculosis* **(** **Table 2**). This segment is specific for the MTB complex, consisting of *Mycobacterium tuberculosis*, *Mycobacterium bovis* and *Mycobacterium simiae* ***.*** IS*6110* is present in multiple copies in *M. tuberculosis* strains and in a single copy in *M. bovis* strains [37], [38]. The nested-PCR method is employed to detect the presence of mycobacterium element. The first pair of primers (P1 and P2) amplifies a 123-bp. The second set of nested primers; (IS-3 and IS-4) partially overlapping P1 and P2 amplifies a 92-bp product **(** **Table 2** **)**. The PCR mixture consisted of 1x PCR buffer (Faststart, Roche), 1 mM MgCl2, 200 µM each of dNTPS, 2 µM of each primer (Sigma), BSA (at a final concentration of 4 µg), 1 Unit of Faststart Taq polymerase (Roche), extracted DNA, and the reaction volume was made up to 10 µl by adding PCR grade water. Amplification steps consisted of an initial denaturation at 94°C for 5 min and 42 cycles of 40 s at 94°C, 1 min at 68°C, and 20 s at 72°C, followed by 5 min at 72°C. For the nested PCR, 1 µl of diluted first-round PCR product was used as the template. Nested amplification was performed using the same thermal profile as before, but applying only 25 PCR cycles in total.

Specificity of the *IS6110* PCR was confirmed by restriction digestion and direct sequencing. For restriction digestion, 4 µl of the PCR products were incubated with 10 U *SalI* (NEB) for 2 hours at 37°C in a water-bath. For direct sequencing, the PCR products were purified using ExoSAP-IT (USB) following the recommended protocol of the manufacturer. Cycle- sequencing was performed with the BigDye terminator sequencing kit (Applied Biosystems). Automatic sequencing was performed on an ABI sequencing model 3100 (Applied Biosystem).
